# The Glycosylphosphatidylinositol biosynthesis pathway in human diseases

**DOI:** 10.1186/s13023-020-01401-z

**Published:** 2020-05-28

**Authors:** Tenghui Wu, Fei Yin, Shiqi Guang, Fang He, Li Yang, Jing Peng

**Affiliations:** 1 Department of Pediatrics, XiangYa Hospital, Central South University, 87 Xiangya Road, Changsha, 410008 Hunan Province China; 2 Hunan Children’s Mental Disorders Research Center, XiangYa Hospital, Central South University, 87 Xiangya Road, Changsha, 410008 Hunan Province China

**Keywords:** GPI-APs, PIG/PGAP genes, Phenotype

## Abstract

Glycosylphosphatidylinositol biosynthesis defects cause rare genetic disorders characterised by developmental delay/intellectual disability, seizures, dysmorphic features, and diverse congenital anomalies associated with a wide range of additional features (hypotonia, hearing loss, elevated alkaline phosphatase, and several other features). Glycosylphosphatidylinositol functions as an anchor to link cell membranes and protein. These proteins function as enzymes, adhesion molecules, complement regulators, or co-receptors in signal transduction pathways. Biallelic variants involved in the glycosylphosphatidylinositol anchored proteins biosynthetic pathway are responsible for a growing number of disorders, including multiple congenital anomalies-hypotonia-seizures syndrome; hyperphosphatasia with mental retardation syndrome/Mabry syndrome; coloboma, congenital heart disease, ichthyosiform dermatosis, mental retardation, and ear anomalies/epilepsy syndrome; and early infantile epileptic encephalopathy-55. This review focuses on the current understanding of Glycosylphosphatidylinositol biosynthesis defects and the associated genes to further understand its wide phenotype spectrum.

## Introduction

Glycosylphosphatidylinositol anchored protein (GPI-AP) consists of glycosylphosphatidylinositol (GPI) anchor and a certain protein. At least 150 different human proteins, including > 40 enzymes [alkaline phosphatase (ALP), 5′-nucleotidase, dipeptidase, and others], several adhesion molecules (contactins, glypicans, CD48, and others), receptors (folate receptors, GDNF receptor alphas, FcγRIIIb, and others), protease inhibitors (RECK), transcytotic transporters (GPIHBP1), and complement regulatory proteins (CD55 and CD59), are anchored via GPI anchor [1] .

**Fig. 1:**
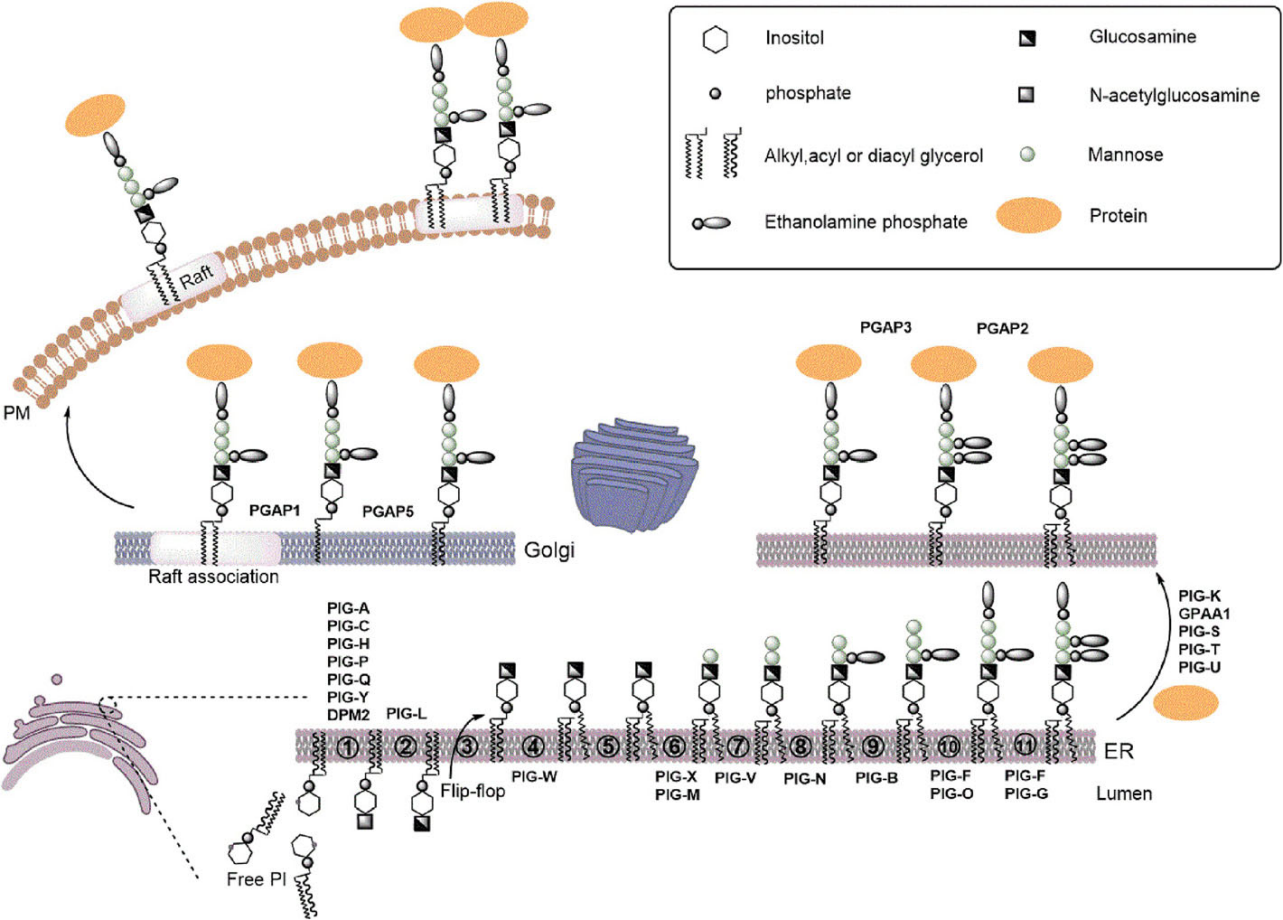
A scheme for the overall GPI-APs biosynthetic pathway, structural remodeling and transport. The whole biosynthetic steps can be divided into three parts: biosynthesis of GPI anchor, attachment of protein and GPI anchor, remodeling of GPI-APs. a) GPI anchor is synthesized in the ER from free PI through 11 steps [transfer of GlcNAc to PI, de-N-acetylation, acylation of the inositol ring, transfer of three mannoses, transfer of three ethanolamine phosphates, currently unknown: GlcN-PI flips from the cytoplasmic side to the luminal side, lipid structure changes from diacyl PI to a mixture of 1-alkyl, 2-acyl PI and diacyl PI in GlcN-(acyl) PI] which involves more than 17 genes, most of them are named PIG genes. The first two steps take place on the cytoplasmic side of the ER, whereas subsequent steps occur on the luminal side. b) The precursor proteins are synthesized independent of the GPI and processed by the GPI-transamidase complex encoded by five genes. c) Post-translational modification after attachment of protein and GPI anchor involving PGAP (post-GPI-attachment to proteins) genes, includes structural remodeling of the glycan and lipid portions of the GPI anchor, removing an acyl chain from the inositol and an EtN-P from Man-2. GPI-APs are then transported to the cell surface through the Golgi, where additional structural remodeling occurs, namely fatty acid

The whole biosynthetic pathway involves at least 16 reactions and more than 20 different proteins encoded by Phosphatidyl Inositol Glycan (PIG) / Post GPI Attachment to Proteins (PGAP) genes (Supp. Table 1). The pathway can be divided into the following three parts (Fig. 1): biosynthesis of GPI anchor, attachment of protein and GPI anchor, and remodelling of Glycosylphosphatidylinositol anchored proteins (GPI-APs). In the first part, the former 10 steps that occur in the endoplasmic reticulum (ER) synthesize the common core structure of GPI anchor composed of a molecule of phosphatidylinositol (PI) and a glycan part that contains glucosamine, three mannoses, and an ethanolamine phosphate [1]. In the second part, the preassembled GPI anchor is transferred to the carboxyl terminus of protein, and this is mediated by transamidase enzyme complex that consists of five components (PIG-K, hGAA1, PIG-S, PIG-T, and PIG-U). After the attachment of the GPI anchor to protein, several modifications occur on the glycan, lipid portions, the inositol-linked acyl chain, and ethanolamine phosphate at the second mannose during transportation to the cell surface from the ER through the Golgi apparatus. When one of the three parts is defective, these proteins have the following two possible outcomes: intracellular degradation or secretion [2].

Biallelic variants in PIG/PGAP genes are responsible for Glycosylphosphatidylinositol biosynthesis defects (GPIBDs) that are associated with broad clinical features, including developmental delay/intellectual disability, seizures, dysmorphic features, and diverse congenital anomalies. To date, PIG/PGAP genes mutations have been reported to cause various GPI deficiency disorders and phenotypes, including multiple congenital anomalies-hypotonia-seizures syndrome (MCAHS); hyperphosphatasia with mental retardation syndrome (HPMRS)/ Mabry syndrome; coloboma, congenital heart disease, ichthyosiform dermatosis, mental retardation, and ear anomalies/epilepsy syndrome (CHIME syndrome) and other GPIBDs. To date, a total of 19 genes have been reported to be responsible for GPI deficiency disorders since the first disease-causing PIG gene was discovered in 2006 [3]. However, in this big gene family, the total number of patients reported from 2006 to the present, was less than 250 patients, and many genes were introduced in case reports, lacking of systematic summary. More than half of patients appeared in the last 5 years. A study that analysed the exome data from 4293 trios showed that PIG/PGAP genes are responsible for only around 0.15% of cases with developmental delay [4].

The main aim of this review is to describe the wide spectrum of GPIBDs caused by PIG/PGAP genes mutations, since other reviews provide a more detailed description of the biosynthesis of GPI-APs [5, 6]. Here, we focus on the current understanding of the PIG/PGAP genes from a human disease perspective. In particular, we provide an overview of GPIBDs, and we review the clinical data as well as the biochemical findings.

## GPI deficiencies caused by somatic mutations

Paroxysmal nocturnal hemoglobinuria (PNH) is an acquired clonal blood disorder characterized by hemolysis, thrombosis, and bone marrow failure, firstly linked to PIGA gene by Takeda et al. in 1993 [7]. The most important pathogenic mechanism is the deficiency of two GPI-APs (CD55 and CD59) caused by somatic PIGA or PIGT gene mutations in the hemopoietic stem cells. The PIGA gene is located in X chromosome, so only the mutation occurs in a male cell or on the active X chromosome in a female cell causes disorder. Instead, PIGT gene in autosomal chromosome, shows loss of function only when two independent somatic mutations or a germline and a somatic mutation occur together [8]. Therefore, PIGA gene mutation is more common than PIGT in PNH. For a patient with hemolysis, the flow cytometry without sequencing will find the GPI-APs deficiency for diagnosis. In this review, we will focus on germline mutations in PIG/PGAP genes.

## GPI deficiencies caused by germline mutations

### MCAHS

In 2011, Maydan et al. described that MCAHS is an autosomal recessive disorder characterised by neonatal hypotonia, lack of psychomotor development, seizures, dysmorphic features, and variable congenital anomalies involving the cardiac, urinary, and gastrointestinal systems, and they first stated that this disorder is caused by a homozygous variant in PIGN [9]. Since then, the other PIG/PGAP genes responsible for MCAHS, including PIGA and PIGT genes, have been discovered [10, 11]. MCAHS caused by PIGN, PIGA, and PIGT genes was named as MCAHS1 (GPIBD3, OMIM #614080), MCAHS2 (GPIBD4, OMIM #316818), MCAHS3 (GPIBD7, OMIM # 615398), respectively, according to the time of the first report.

#### PIGN

EtNP transferase 1, encoded by PIGN gene, is a crucial enzyme in the eighth step of the GPI-anchor biosynthetic pathway, transferring ethanolamine phosphate to the first alpha-1,4-linked mannose of the glycosylphosphatidylinositol precursor of GPI-anchor [12].

MCAHS1 is an autosomal recessive disorder. To date, a total of 30 patients have been reported with PIGN gene mutation. Twenty patients were diagnosed with MCAHS1 and 10 patients with Fryns syndrome (FS).

As the name MCAHS suggests, the crucial clinical manifestations include multiple congenital anomalies (14/20), hypotonia (20/20), epilepsy (19/20), development delay (20/20), and dysmorphic features (13/18). Other shared clinical features include nystagmus (7/20), deep plantar groove, swallowing problems, and feeding difficulties. Dysmorphic features include bitemporal narrowing, long philtrum, micrognathia, hypertelorism, depressed nasal bridge, small nose, thin upper lip, low-set ears, and overfolded helices. Several recent reports have proposed a genotype–phenotype correlation suggesting that congenital anomalies are linked with truncating variants [13–16]. However, in general, congenital anomalies are common in MCAHS1, but they are not multiple in one patient. Gastroesophageal reflux is the most common anomaly. In addition, urinary system, including vesicoureteral reflux or duplicated collecting system is relatively common. Significantly, most seizures are refractory except for two complex partial seizures, one of which is treated with levetiracetam and topiramate [16, 17]. The onset age of seizure is within the first year of life (20 d–9 m). There was a case of a boy with late onset seizure at the age of 11 years; however, he had a severe developmental delay and he died at the age of 14 years [15]. Intractable epilepsy and complication rather than congenital anomalies of the cardiovascular system or urinary system are fatal for patients; hence, a genotype–phenotype correlation associated with seizure and developmental level is needed for better estimation of the prognosis.

Ten patients with FS confer a worse prognosis. Pregnancy loss by miscarriage or stillbirth in 6 patinets, and the other 4 patients almost died in the first month of life. Congenital diaphragmatic hernia (CDH) is a major feature, occurring in 7 patients and the most common cause of death is pulmonary hypoplasia. Alessandri et al. described the clinical characteristics, including polyhydramnios (6/10), facial dysmorphism (8/10), brachytelephalangy (8/10), orofacial cleft (7/10), cardiovascular malformations (6/10), Genitourinary anomalies (65/10) [18].

To date, 20 point mutations (Fig. 2), 2 frameshift and 3 microdeletions have been discovered in 19 families. Most PIGN gene mutations (18/22) are involved in both the phosphodiesterase and PigN domains. Functional analysis showed that red blood cells displayed similar amounts of CD59 in the affected persons and healthy controls. However, CD59, CD16, CD24, CD18, and FLAER in granulocytes showed reduction compared to that in healthy controls [16, 17, 19, 20].

#### PIGA

PIGA gene encodes PIG-A, a catalytic subunit of GPI-GlcNAc transferase (GPI-GnT) [21], which is involved in the first step. PIG-A has one transmembrane domain near the C-terminus, and two highly conserved domains PigA and Glycos_transf_1 [22].

Somatic PIGA gene mutations had been identified in many patients with PNH, and it had been proposed that germline PIGA gene mutations were lethal due to an early block in embryogenesis before the development of the mesoderm and the endoderm, resulting from loss of GPI anchored co-receptors involved in BMP4 signalling [23–26]. In 2012, Johnston et al. first described the pedigree of a family with MCAHS2 due to a hypomorphic germline PIGA gene mutation [10]. MCAHS2 is the only GPIBD inherited in X-linked recessive heredity. To date, 31 cases with germline PIGA gene mutations have been reported, and all male patients. Among them, most were diagnosed with MCAHS and the other patients were diagnosed with Ferro-Cerebro-Cutaneous Syndrome (FCCS) or Early infantile epileptic encephalopathy, but there is a significant overlap between these phenotypes.

MCAHS2 is associated with hypotonia (19/26), epilepsy (27/27), and dysmorphic facial features (16/27). Tonic (6/27), myoclonic (5/27), spasm, atonic, tonic-clonic seizures, atypical absence and status epilepticus were observed in MCAHS2. Early myoclonic encephalopathy, Ohtahara syndrome, and West syndrome were commonly found in MCAHS2 and EEG revealed a suppression-burst or hypsarrhythmia pattern. Brain MRI showed a thin corpus callosum; delayed myelination; a small cerebellum; cortical atrophy; and restricted water diffusion in the brainstem, basal ganglia, or cerebellum, without any specific finding. Some patients had abnormalities of the heart, liver, and kidneys, as well as deafness and visual impairment. Seven patients had high serum ALP.

In 2014, Kato et al. first proposed that the phenotypic consequences of PIGA gene mutations can be classified into two types, severe and less severe [27]. Severe forms show myoclonus and asymmetrical suppression-burst on EEG, multiple anomalies with a dysmorphic face, and delayed myelination with restricted diffusion patterns in specific areas. The less severe form presents with intellectual disability and treatable seizures without facial dysmorphism, which correlate with the degree of PIG-A activity reduction caused by the mutations. Five less-severe patients were quite consistent with the classification proposed by Kato et al. [27–30]. Focal tonic or tonic–clonic provoked by fever or viral illness, no myoclonic seizures, were manifested. The onset age of seizure is between 5 to 7 m, and the seizure frequency decreases significantly at the age of 15 to 24 m, without any apparent improvement in intellectual disability. However, the patient with facial dysmorphism reported by Soden et al. and Low et al. later weakened the general value of this hypothesis [31, 32].

Eighteen different PIGA gene mutations have been described. Among them, c.1234C > T (p.R413X) accounts for a large proportion of PIGA gene mutations, and most of the mutations are located in the PigA domain. GPI-APs show decreased expression on granulocytes, T cells, and B cells, and normal erythrocyte expression, which may explain the absence of haemolysis in germline mutations.

#### PIGT

PIGT encodes a subunit of a heteropentameric transamidase complex catalysing the attachment of proteins to the GPI anchor. In the absence of PIG-T, GPAA1 and PIG-K were expressed lowly, whereas the expression levels of PIG-S was normal, indicating that PIG-T is critical for the stability of GPAA1 and PIG-K [33].

To date, thirteen patients from six families, including six males and seven females, have been diagnosed with PIGT-associated MCAHS3 (OMIM * 610272). PIGT mutations present with severe intellectual disability (13/13), refractory seizures (7/13), facial dysmorphism (11/13), and hypotonia (10/13). Multiple congenital anomalies include heart defect (6/10), nephrocalcinosis (6/11), skeletal abnormalities (7/11), which provide important basis for clinical diagnosis. There were no cardiovascular abnormalities. In addition, impaired vision (8/13) and feeding difficulty (5/13) are common. Myoclonic, tonic and tonic-clonic seizures were observed in different PIGT gene mutations. Facial dysmorphism, includes a high forehead, frontal bossing, bitemporal narrowing, a short nose with depressed nasal bridge, a wide open mouth, tented lips, high-arched palate, and low auricular position. Together, in all previous studies, seven patients with MCAHS3 had low serum ALP in contrast with the other phenotypes of PIG/PGAP genes, including PIGV, PIGO, PGAP2, PGAP3, PIGW, and PIGY.

Nine different PIGT gene mutations (Fig. 4, including one nonsense, two frameshift, and six missense) have been reported. The PIGT variant causes significant reductions in the level of GPI-anchored proteins, including CD14, CD16, CD18, CD24, CD55, and CD59, which has been observed in six of the 12 reported patients. However, the mRNA and protein expression levels of PIGT gene mutation were increased in studies by Lam et al. [34] and Yang et al. [35]. One potential explanation is that the mutation causes loss of function, and therefore, the mRNA increase may have been contributed by the negative feedback regulation.

### HPMRS / Mabry syndrome

HPMRS is an autosomal recessive disorder characterised by intellectual disability and elevated levels of serum ALP (alkaline phosphatase is a GPI- AP), and it is often accompanied by seizures, facial dysmorphism, and various anomalies such as brachytelephalangy. To date, six genes involved in GPI biosynthesis, including PIGV, PIGO, PGAP2, PGAP3, PIGW, and PIGY, have been reported to be responsible for HPMRS (namely HPMRS1–6 orderly). High serum ALP is a feature of HPMRS, which is different from the other GPIBDs.

ALP is important for neuronal development and plays a major role in vitamin B6 (pyridoxine) metabolism [36]. Alpl knockout mice develop intractable seizures that are responsive to administration of vitamin B6. Kuki et al. described a 9-year-old male with intellectual disability and vitamin B6 responsive epilepsy caused by PIGO gene mutation [37].

#### PIGV

GPI-mannosyltransferase II, encoded by PIGV gene, is involved in the seventh step. PIGV gene mutations disrupt the addition of the second mannose to the GPI anchor that is necessary to tether ALP at the plasma membrane [37], leading to HPMRS1 (GPIBD2, OMIM # 239300).

To date, 23 patients with a PIGV gene mutation in 18 families have been reported. The core features of HPMRS1, such as hyperphosphatasia and mental retardation, were found in all patients. The level of ALP fluctuated from 1.54- to 17.20-times (median, 3.54-times) of the upper limit of the normal range. No speech and no walking or working with support development was noted in most patients. Brachytelephalangy (16/18) is another feature of PIGV-related GPIBD, and this characteristic differentiates it from the other HPMRSs. In addition, hypotonia (16/21), seizures (17/23), and dysmorphism (21/21), including apparent hypertelorism, long palpebral fissures, broad nasal bridge/tip, and tented mouth, are the common features, which are consistent with the other GPIBDs.

Seventeen different PIGA gene missense mutations have been described. Among them, c.1022C > A (p.A341E) accounts for a large proportion of PIGV gene mutations. GPI-APs, besides ALP, FLAER and CD16 on leukocytes were detected with a marked decrease [38]. Although a genotype–phenotype correlation remains unclear due to the limitation of relevant data, high ALP, brachytelephalangy, and some common symptoms of GPIBDs are enough to take HPMRS into consideration.

#### PIGO

PIGO encodes a catalytic component of GPI- EtNP transferase III that functions in the tenth step by transferring phosphatidylethanolamine (PE) to the third mannose (Man3) of the GPI core. PIG-O is stabilized by PIG-F [38].

Eighteen patients with a PIGO gene mutation have been identified. Initially PIGO gene mutations were associated with the HPMRS2 (GPIBD6, OMIM # 614749); however, the clinical spectrum spans from the HPMRS2 phenotype to severe infantile onset epileptic encephalopathy. An overlap between these phenotypes includes seizure (12/18), developmental delay (18/18), and hypotonia (12/13), which are also common symptoms in GPIBDs. In addition, gastrointestinal anomalies (13/18) including anal atresia, Hirschsprung and brachytelephalangy (15/18), as the characteristic manifestations, will play a great role in the diagnosis of HPMRS2. Hyperphosphatasia (10/13) can be found in HPMRS2, although marked elevations of ALP are not mandatory in patients with PIGO gene [39, 40]. Less commonly reported symptoms include dysmorphism (11/17), hearing loss (9/18). Congenital anomalies including tetrology of Fallot, vesico-ureteral reflux and cryptorchidism are not remarkable. A genotype-phenotype relation suggesting that mutations in the ALP-like core domain (PigO domain) cause severe neurological presentation has been proposed by Nakamura et al. [39]

Reported PIGO gene mutations include 25 mutations (including nonsense, frameshift, missenses, and splice site) and they cause variable decrease in GPI-APs levels in blood cells. CD16 and CD24 were normally expressed on the cell surface [41]. On the contrary, decreased FLEAR, CD16, and CD24 expression on the granulocyte was noted in another report [42].

#### PGAP2

PGAP2 gene refers to the lipid remodelling step of GPI-anchor maturation, and it is required for stable expression of GPI-APs on the cell surface.

PGAP2 gene mutation is responsible for HPMRS3 (GPIBD8, OMIM # 614207), which presents with varying degrees of mental retardation and elevated ALP. Less commonly reported symptoms include epilepsy (10/15), hypotonia, microcephaly and dysmorphism. Fifteen patients and 10 missense mutations, 6 of which were homozygous, have been described. The expression levels of GPI-APs (CD59 and CD55) were associated with the location of mutation; likewise, the phenotype severity correlates with conservation level of this position [43]. However, identification of other patients with PGAP2 gene mutations is required to confirm this observation.

#### PGAP3

PGAP3 gene is involved in fatty acid GPI remodelling that is critical for proper association between GPI-APs and lipid rafts.

In 2014, Howard et al. identified homozygous or compound heterozygous mutations of the PGAP3 gene in five patients from three families with HPMRS [44], and thus, they named it HPMRS4 (GPIBD10, OMIM # 615716). All patients showed global developmental delay, hypotonia, high ALP fluctuating from 1.23- to 5.00-times the upper limit of normal (median, 2.42-times the upper limit of normal), and other shared clinical features including epilepsy (generalized tonic-clonic, myoclonic), dysmorphism (hypertelorism, broad nasal bridge, small nose, broad nasal tip, short nose, tented upper-lip vermilion, large and fleshy ear lobes, and cleft palate.), without brachytelephalangy usually caused by PIGO and PIGV genes. It is noteworthy that the median age of onset of seizures is 4 y (1.5–12 y), and the median age of last assessment is 8 years (2–17 y), which suggests a better outcome than that for the common PIG genes. However, the reduction of GPI-AP levels is variable, and further study is needed [44].

#### PIGW

PIGW gene encodes a 504-amino-acid inositol acyltransferase that acts in the third step of GPI biosynthesis. Inositol acylation is critical for the attachment of bridging ethanolamine phosphate to the third mannose [44].

In 2014, Chiyonobu et al. discovered a PIGW gene variant in a patient with intractable seizures and developmental delay, mild dysmorphic facial features (broad nasal bridge and tented upper lip), inguinal hernia, typical hypsarrhythmic pattern in EEG, and constantly elevated ALP (2000 U/L) [45], which led to the discovery of HPMRS5 (GPIBD11, OMIM # 616025). Consequently, Hogrebe et al. presented two patients with normal serum ALP, remarkably different from the phenotype of the former [46]. Clinical manifestations lacking of specificity, make it difficult to identify patients with HPMRS5.

The reduction in GPI-AP levels depends on the cell type and species. Flow cytometry by Chiyonobu et al. revealed that CD59 and FLAER on lymphocytes and CD14 on monocytes were reduced to half of that in controls. However, CD16, CD24, and CD66c on granulocytes showed subtle changes in a study by Hogrebe et al. [46]. To date, there are three patients from two families, and therefore, a genotype-phenotype relation for PIGW gene mutations needs to be further elucidated.

#### PIGY

PIGY gene encodes the smallest subunit of GPI-GnT, which is directly associated with the catalytic subunit PIG-A. PIG-Y may regulate the catalytic action of PIG-A, but it is not essential for GPI-GnT activity [47].

In 2015, Ilkovski et al. presented the first report of PIGY gene mutation [48]. To date, only four patients (three females, one male) from two families have been diagnosed with HPMRS6 (GPIBD12, OMIM # 616809), and they presented with characteristics often seen in GPIBDs. All four patients had global developmental delay or developmental regression and ALP was elevated in two females from family 1 (2.84-times the upper limit of normal). Functional study showed significantly reduced levels of GPI-APs (CD55 and CD59) on the surface of skin fibroblasts [48].

### CHIME syndrome

CHIME syndrome (GPIBD5, OMIM # 280000) is also known as Zunich neuroectodermal syndrome. In 1983, Zunich et al. first described this new syndrome of congenital ichthyosis with neurologic abnormalities [49], and it is an extremely rare autosomal recessive multisystemic disorder. In 1995, Shashi et al. suggested that the disorder should be called CHIME syndrome, as it is clinically characterized by colobomas, congenital heart defects, early onset migratory ichthyosiform dermatosis, mental retardation (intellectual disability), and ear anomalies (conductive hearing loss). Alternatively, the ‘E’ could stand for seizures (epilepsy) [50]. Ng et al. identified the causative gene PIGL in 2012 and that CHIME syndrome is inherited in an autosomal recessive manner [51]..

PIGL gene is involved in the second step of GPI biosynthesis. PIG-L contains a transmembrane domain (a.a2–22) and the core de-N-acetylase domain (a.a44–168). To date, PIGL mutations have been reported in seventeen individuals mainly with CHIME syndrome (high ALP but no ichthyosiform dermatosis in four individuals made the presentation more suggestive of HPMRS) [4, 52, 53]. Resembling other GPIBDs, intellectual disability, seizure, and dysmorphic features (sparse, fine hair, ocular hypertelorism, flat/broad nasal root, ears with overfolded helices) are observed in all CHIME syndrome, and congenital anomalies such as heart defects and renal abnormalities are relatively common. The characteristics for differentiation of CHIME syndrome from other GPIBDs are ichthyosiform dermatosis (11/11) and ear anomalies (9/9); in addition, coloboma was markable in six patients reported by Ng et al. However, the clinic features, elevated ALP and brachytelephalangy, in the absence of ichthyosiform dermatosis, coloboma, heart defect and ear anomalies, demonstrated HPMRS. Undoubtedly, it is not clear why some PIG-L defects are particularly associated with HPMRS.

So far 22 variations have been reported in PIGL gene, and most of the mutations affect the core de-N-acetylase domain (7/8). Extensive biochemical analysis revealed a clear decrease in CD59 and FLAER (a primary fibroblast line), CD24 and CD16 (granulocytes), and CD14 (monocytes).

### Early infantile epileptic encephalopathy-55 (EIEE55)

EIEE55 (GPIBD14, OMIM # 617599) is caused by a compound heterozygous mutation in the PIGP gene. PIG-P is an essential subunit of GPI-GnT, which interacts with PIG-A and PIG-Q [6]. In 2017, Johnstone et al. first reported the compound heterozygous variants of PIGP gene in two siblings who presented with early-onset refractory seizures, hypotonia, and profound global developmental delay. Functional studies with patient cells showed reduced mRNA levels, and an associated reduction in GPI-anchored cell surface proteins (CD16, CD55, and CD59) [54].

### GPIBD9

PGAP1 catalyses the inositol deacylation of GPI in an early step during remodelling of GPI-APs biosynthesis, cleaving the acyl chain from the inositol, which is essential for the generation of mature GPI capable of attachment to proteins [55].

In 2014, the first PGAP1 gene was described in a floppy infant with developmental delay and severe intellectual disability [56]. Ten patients from six families have been described so far, and most of them showed developmental delay/intellectual disability (9/10) and hypotonia (8/10), as manifested above. Significantly, only two individuals presented with seizure and one patient presented with abnormal EEG, and those patients without seizures did not show a better motor or intellectual development. Otherwise, microcephaly (7/8) and feeding problems (6/8) are more prominent than in other GPIBDs. Other shared presentations included dyskinetic movements (6/10) and dysmorphic features. Brain imaging showed global brain atrophy (5/9) and defective myelination (4/9).

Murakami et al. demonstrated that the defect of PGAP1 did not decrease the expression of GPI-APs on B lymphoblastoid cells, but it led to an abnormal structure and altered biochemical properties [56].

### GPIBD13

PIG-G, which forms a complex with PIG-F, is a catalytic component of GPI ethanolamine phosphate (GPI-EtNP) transferase II in the eleventh step. PIG-G is stabilized by PIG-F, and it competes with PIG-O for binding to PIG-F [57].

Seven affected individuals from four families have been diagnosed with a PIGG gene mutation. The common symptoms were hypotonia, severe developmental delay, seizures (6/7), cerebellar ataxia, and cerebellar hypoplasia, with or without mild facial dysmorphology. Particularly, seizures in two patients slowly diminished from the age of 6 months and disappeared completely at around 9 months, although the median age at seizure onset of all 6 patients with seizures was 4 m (2–10 m). Also, all six patients survived from 7 to 24 y (the median age was 11 years at the last investigation), although they had significant intellectual disability and seizures. The flow cytometry result showed totally different findings in the two available studies [58, 59]; therefore, more related data and further study are needed.

### GPIBD15

GPIBD15 is caused by a mutation in the GPAA1 gene (603048). hGAA1 (glycosylphosphatidylinositol anchor attachment 1) is an essential component of the transamidase attaching the GPI anchor to the C terminus of precursor proteins in the endoplasmic reticulum. Most individuals presented with global developmental delay, hypotonia, early-onset seizures, cerebellar atrophy, and osteopenia. Flow-cytometry showed decreased GPI-APs in leukocytes (FLAER, CD16, and CD59) or fibroblasts (CD73 and CD109), [60].

### GPIBD16/MRT62

Glycosylphosphatidylinositol biosynthesis defect-16 (GPIBD16) is caused by a homozygous or compound heterozygous mutation in the PIGC gene (601730). PIG-C is an essential subunit of GPI- GnT [6]. In 2017, three PIGC gene mutations were identified in three patients from two unrelated families who suffered from global developmental delay, severe intellectual disability, and drug-responsive seizure disorder. A constant decrease in the CD16 level in granulocytes, and a moderate decrease in CD14, CD55, CD59, and FLAER levels were detected [61].

### GPIBD17

In 2018, a PIGH gene variant was discovered in a boy with epilepsy, microcephaly, and behavioural difficulties. Nguyen et al. subsequently reported a patient with developmental delay and autism, but without an epileptic encephalopathy. Phenotypic variability is expected to increase with ongoing reports of these patients. Together with five other subunits, PIG-H is an essential component of GPI- GnT, an enzyme that is critical in the first step of GPI-anchor biosynthesis [22]. To date, all five subunits of GPI-GnT have been clarified as disease-causing genes. The GPI-APs (CD59, DAF, CD16, CD55 and uPAR) evaluated by flow cytometry and Western blotting showed a decrease.

### Unclassified

PIG-Q stabilizes the enzyme complex in the first step of GPI biosynthesis. Watanabe et al. demonstrated that PIG-Q is not an essential component of GPI-GnT because PIGQ-defective cells have some surface GPI-AP expression [62]. However, a recessive mutation in PIGQ gene have been reported in an Ohtahara Syndrome patient and a patient with profound developmental delay, dysmorphic features, hypotonia, vision problems, gastrostomy tube dependency [63, 64]. The transcriptional level, the expression of mutant protein, and the GPI-APs (CD59) detected by qPCR, western blotting and flow cytometry, respectively, were greatly decreased.

In 2006, Almeida et al. identified a PIGM gene mutation in 7 individuals with venous thrombosis and seizures. Absence seizures and portal vein thrombosis were the main characteristic and Pode-Shakked et al. suggested that the PIGM mutation be considered in the differential diagnosis of infantile- or early-onset stroke [65]. The surface expression of FLAER, CD59, and CD24 in the granulocytes showed a clear reduction; however, CD59 in the erythrocytes and platelets was preserved.

## Discussion and conclusions

### Unclear genetic bases and phenotypes

Although most of the genes are well characterised, the regulation of GPI biosynthesis remains unclear. In the biosynthetic pathway, the genetic bases of two steps; step 3, in which GlcN-PI flips from the cytoplasmic side to the luminal side, and step 5, in which the lipid structure changes from diacyl PI to a mixture of 1-alkyl, 2-acyl PI, a major form, and diacyl PI, and a minor form of GlcN-(acyl) PI, are currently unknown.

To date, nineteen genes involved in the biosynthesis of GPI-APs have been reported, and additional genes (PIGB, PIGF, PIGK, PIGS, PIGU, and PIGX) involved in this pathway are awaiting discovery:

- Remaining genetic conditions are so rare that a longer time period and a larger cohort are needed to identify the genes.
- Individuals with variants in other GPI genes might not present with a series of characteristic clinical features like developmental delay, seizures, and facial dysmorphism, or are embryonically lethal.
- The remaining six genes may not be mandatory in the GPI-anchor synthesis pathway.
- Data filtering standards that excluded variants may be a disease-causing point.

Out of the four possibilities, the latter two seem more likely than the other two. For example, PIG-X and PIG-F only provide a stability function of PIG-M and PIG-O [66, 67], whose deficiencies may not affect the normal function of PIG-M and PIG-O, and the other four GPI transamidase components can still form a complex in the absence of PIG-U [6]; or the patients with these genes presented with mild symptoms that did not need genetic testing. In addition, a low-coverage variant is often considered low-confidence and filtered out in the early stages of analysis; however, it may be a new disease-causing gene, such as PIGH gene, discovered by Pagnamenta et al. in 2018.

### Correlation between the genotype and the phenotype

GPI-APs can be cleaved by GPI-specific phospholipases or other enzymes targeting GPI glycan or the peptide part, and proprotein can be released [68]. When GPI anchor biosynthesis is defective, these enzymes cannot function correctly, leading to the following two possible outcomes: intracellular degradation or secretion [2]. Murakami et al. proposed that the degree of serum ALP levels is partly dependent on the position of the gene mutation in the GPI biosynthesis pathway: gene (PIGA, PIGL, PIGM, PIGN, and PIGT) mutations occurring in the early GPI-anchor synthesis and the attachment of proteins to the GPI anchor result in primary reduced surface levels of GPI-APs because of increased intracellular degradation of incomplete GPI anchor; therefore, these genes are not associated with high serum ALP. Mutations in PIGV, PIGO, PGAP2, PGAP3, and PIGW, which affect late GPI-anchor synthesis, are associated with high serum ALP, because GPI transamidase can recognize the currently incomplete GPI anchor and cleave the GPI attachment signal, resulting in reduced GPI-AP surface levels and increased secretion into the extracellular space. However, HPMRS was subsequently discovered in the early steps involving PIGY, which was in contrast with what was predicted previously. Although, PIGY is an exception, possibly due to the function that PIG-Y regulates the catalytic action of PIG-A, but it hardly affects the GPI-GnT activity. In addition, we concluded that all genotypes and phenotypes are involved in the GPI biosynthesis pathway and discovered that ALP was elevated in six patients with a PIGA mutation. So far, high ALP was noted in the 1st, 2rd, 4th, 7th, and 10th step and the last remodelling step. Therefore, whether serum ALP is elevated or not does not seem to be linked with the steps. Further reports are required to clarify this matter.

Comparison of clinical findings between different PIG/PGAP genes, uncovered that neurological symptoms, such as developmental delay, seizure, hearing loss, and brain MRI abnormalities, are similar. In these patients, the median age at seizure onset ranges from 3 m ~ 7y8m, and it is not linked with the physical localization of these different PIG/PGAP genes (Table 1). However, the prognosis is quite different. The phenotype of MCAHS (caused by PIGA, PIGN, and PIGT) is much severe than the other GPIBDs. Twelve patients (38.7%) and eighteen patients (60%) with PIG-A deficiency and PIG-N deficiency, respectively, died early in life, whereas only one patient with PIG-L and PIG-V deficiency died, and none of the patients with PIG-G deficiency died. It has been suggested recently that mutations in proteins that play a role later in the GPI anchor biosynthesis do not always result in a less severe clinical outcome when compared with mutations in proteins that function early in this pathway, such as PIG-L (early) and PIG-N (later). Significantly, PGAP mutations indicate a much better prognosis.

**Table 1:** Comparison of clinical findings between different PIG/PGAP genes (PIGA, PIGL, PIGV, PIGM, PIGN, PIGO, PIGG, PIGT, PGAP1, PGAP2, PGAP3)

Genes	Steps	Related diseases	Total number of patients	Median age at seizure onset	Median age at death	Number of deaths
PIGA	1	MCAHS2	31	6 m	2.78 m	12
PIGL	2	HPMRS5	17	5 m	1y	1
PIGM	6	GPIBD1	7	3.75y	5.5y	2
PIGV	7	HPMRS1	23	7 m	10y	1
PIGN	8	MCAHS1	30	3 m	19d	18
PIGO	10	HPMRS2	18	1.83y	1.75y	8
PIGG	11	MRT53	7	4 m	–	0
PIGT	12	MCAHS3	13	5 m	7y8m	5
PGAP2	Remolding	HPMRS3	15	7y	–	0
PGAP3	Remolding	HPMRS4	33	4y	–	0
PGAP1	Remolding	GPIBD9	10	3 m^a^	–	0

^a^Only one patient’s onset age is available

### Diagnosis and treatment

GPIBDs manifest heterogeneous clinical phenotypes. Most of the patients present with a series of clinical features like developmental delay, seizures, and facial dysmorphism, which is insufficient to identify patients with potential GPI deficiency. When the patient has abnormal ALP at the same time, HPMRS should be taken into consideration. Exome sequencing is the diagnostic procedure of choice in similar patients. The test of GPI-AP quantification in blood granulocytes of patients can help to evaluate the effect of certain mutations on GPI synthesis. However, GPI-APs (such as ALP, CD59, and FLAER) do not seem to be adequately reliable biomarkers as the overall amount of GPI-APs was normal in certain mutations. In the absence of a more sensitive biomarker, children with GPI deficiency might be missed. It needs to be further elucidated how certain mutations influence GPI-synthesis and protein anchoring in different tissues.

At present, symptomatic treatment is the only approach, and there are no effective antiepileptic drugs. Oral or intravenous use of vitamin B6 (20–30 mg/kg) in HPMRS could improve electroencephalography or control epilepsy as ALP plays an important role in the synthesis of vitamin B6 [37]. In addition, a case report mentions that a ketone diet is effective in children with a PIGA gene mutation [30].

By accumulating more genetic, biochemical, cell biological, and clinical information from many more cases of GPIBDs, we will be able to achieve a better understanding of the mechanistic bases of various symptoms, improved ways for diagnosis, and hopefully useful treatment measures.

## Supplementary information

**Additional file 1: Supplementary Table S1.** The genotype and the clinical data of PIG/PIGP genes.

## Data Availability

Not applicable.

## Abbreviations

ALP: alkaline phosphatase
CDH: congenital diaphragmatic hernia
CHIME: Mabry syndrome; Coloboma, congenital heart disease, ichthyosiform dermatosis, mental retardation, and ear anomalies/epilepsy
EIEE55: early infantile epileptic encephalopathy-55
ER: endoplasmic reticulum
FS: Fryns syndrome
GlcN: N-acetylglucosamine
GPAA1: glycosylphosphatidylinositol anchor attachment 1 (denotes gene name)
hGAA1: Glycosylphosphatidylinositol anchor attachment 1 protein
GPI: Glycosylphosphatidylinositol
GPI-AP: glycosylphosphatidylinositol anchored protein
GPI-APs: glycosylphosphatidylinositol anchored proteins
GPIBDs: Glycosylphosphatidylinositol biosynthesis defects
GPI-EtNP: Glycosylphosphatidylinositol ethanolamine phosphate
GPI-GnT: GPI-GlcNAc transferase
HPMRS: hyperphosphatasia with mental retardation syndrome
MCAHS: multiple congenital anomalies-hypotonia-seizures syndrome
PGAP: Post GPI Attachment to Proteins
PI: phosphatidylinositol
PIG: Phosphatidyl Inositol Glycan
PIGA: Phosphatidylinositol N-acetylglucosaminyltransferase subunit A (gene name without a dash, all else follows)
PIG-A: Phosphatidylinositol-glycan biosynthesis class A protein (protein name indicated with a dash, all else follows)
PNH: Paroxysmal nocturnal hemoglobinuria
